# Impacts of Hospital Payment Based on Diagnosis Related Groups (DRGs) with Global Budget on Resource Use and Quality of Care: A Case Study in China

**Published:** 2019-02

**Authors:** Suwei YUAN, Wenwei LIU, Fengqing WEI, Haichen ZHANG, Suping WANG, Weijun ZHU, Jin MA

**Affiliations:** 1. School of Public Health, Shanghai Jiao Tong University School of Medicine, Shanghai, China; 2. China Hospital Development Institute, Shanghai Jiao Tong University, Shanghai, China; 3. School of Philosophy, Law and Political Science, Shanghai Normal University, Shanghai, China; 4. Department of Quality Control, Longhua Hospital, Shanghai University of Traditional Chinese Medicine, Shanghai, China

**Keywords:** Diagnosis-related-groups (DRGs), Global budget, Hospital payment reform, China

## Abstract

**Background:**

China has implemented numerous pilots to shift its hospital payment mechanism from the traditional retrospective cost-based system to prospective diagnosis-related-group (DRG) -based system. This study investigated the impact of the DRG payment reform with global budget in Zhongshan, China.

**Methods:**

A total of 2895 patients diagnosed with acute myocardial infarction (AMI) were selected from local two largest tertiary hospitals, among which 727 were discharged prior to the payment reform and 2168 afterwards. Difference-in-difference (DID) regression models were used to evaluate the policy effects on patients’ percutaneous coronary intervention (PCI) use, hospital expenditures, in-hospital mortality, and readmission rates within 30 days after discharge.

**Results:**

Patients’ PCI use and hospital expenditures increased quickly after the payment reform. With patients with no local insurance scheme as reference, PCI use for local insured patients decreased significantly by 4.55 percent (95 percent confidence interval [CI]: 0.23, 0.72), meanwhile the total hospital expenses decreased significantly by US$986.10 (b=−0.15, P=0.0037) after reform. No changes were observed with patients’ hospital mortality and readmission rates in our study.

**Conclusion:**

The innovative DRG-based payment reform in Zhongshan suggested a positive effect on AMI patient’s cost containment but negative effect on encouraging resource use. It had no impacts on patients’ care quality. Cost shifting consequence from the insured to the uninsured was observed. More evidence of the impacts of the DRG-based payment in China’s health scenario is needed before it is generalized nationwide.

## Introduction

Payment mechanisms create incentives to influence behaviors of both providers and demanders in the health system (1). Diagnosis-related-groups (DRGs) is tested as a useful tool to save medical costs and increase hospital efficiency through decreasing avoidable health services and improving productivity (2, 3). Meanwhile some evidence also indicated it might compromise the quality of care (1, 4). Owing to its superiority in bending the upward cost curve and releasing the government’s financial pressure, this payment method has been adopted worldwide to reshape hospitals’ funding mechanisms (2, 5, 6).

In 2009, the Chinese government announced to reform its hospital payment mechanism from the retrospective cost-based systems to prospective DRG-based system, with the aim of providing its population with affordable health services (7). In 2011, the National Development and Reform Commission (NDRC) issued a reference list of 104 diagnoses/procedures to guide local governments’ DRG pilots (8). And in 2017, this list expanded to 320(9).

Against this background, many local governments had initiated different kinds of DRG payment pilots (10). 20 of the 32 provinces in mainland China had implemented some kinds of DRGs till 2016(10). However, most of these payment reform models were still in its infancy and piloted in a partial health system (11, 12). These halfway reforms made it difficult to identify the comprehensive effect of the payment reform arrangements in a whole health system (11). Zhongshan, a city in the southeast of China, pro-actively adopted an innovative DRG system together with global budget to pay for its inpatient services in all public hospitals since July 1, 2010.

### Hospital Payment System Reform In Zhongshan

Zhongshan established its social health insurance scheme in 1999. At the beginning, fee-for-service (FFS) was adopted to pay for all health care, which resulted in a rapid increase in hospital expenditures in the following three years. In 2002, the local health insurance administration department explored a special capitation-like retrospective payment method to replace the FFS system and formally carried out in 2004. Under this system, all the insured inpatients were reimbursed depends on their insurance claimed expenses. The middle 95 percent cases were reimbursed through the capitation-like system for the same fixed price, regardless of their diagnoses, comorbidities, or services actually used. The rest highest and lowest 2.5 percent cases were reimbursed through the original FFS approach.

However, a rapid growth in health volumes and inpatient expenses was observed during 2004 to 2009. The inpatient visits had increased by 52 percent during this period, nearly tripled the average provincial level of 19 percent for the same period. And the fiscal deficit ratio of the insurance fund reached to 35.6 percent in 2009. Additionally, due to the great variation of care costs among different cases, this kind of payment system caused frequent conflicts between the insurance management department and the hospital directors.

To address the above problems, Zhongshan pro-actively launched its hospital payment reform combining DRGs with global budget in 2010. Under the new system, all inpatient cases were classified into different payment groups based on two factors: 1) patients’ primary diagnoses, which were identified by the ICD-10 code (International Classification of Diseases, 10^th^ revision); and 2) procedure information. There were four basic procedure categories: medical treatment, traditional operations, minimally invasive surgery and interventional therapy. Through this approach, each payment group is defined with a diagnostic group together with a specific procedure category. A total of 4630 DRGs was included in the latest version of the DRG system in Zhongshan, with a cost weight assigned to each DRG. The cost weight reflects the average claimed expense of patient care compared with other DRGs. The payment rate for a specific DRG was calculated as its cost weight multiplied by the base payment rate. The base payment rate equals the overall budget available for the DRG payment system (approximately 73 percent of the social insurance expenditures) divided by the total weights of all DRGs in the same period. It’s the same for all DRGs during a certain period (usually a fiscal year). The base payment rate was determined at the end of the fiscal year and the final reimbursement amount was calculated for each hospital.

Zhongshan payment reform was unique in three aspects. First, it is a comprehensive reform project that implemented in the whole local health system. Patients reimbursed through the new system accounted for 98 percent of all the insured cases. Second, besides the diagnosis factor, procedure factor was considered for patient classification. Patients were classified into different groups more reasonably and acceptable for practice. The last but most important, the DRG payment rates in Zhongshan were determined flexibly with global budget, which were set to control both system and individual expenditures. Actually the global budget and DRG-based payment are the two main methods that are piloting in China. But they are implemented separately to contain hospital-level and individual-level expenses in most places. In this circumstance, it’s hard to give full play to their strengths in paying for inpatient services. Zhongshan innovatively merged the two payment methods into one composite payment system and united the patient’s cost containment with hospital cost containment.

The main purpose of this study was to investigate the effects of the hospital payment reform in Zhongshan on patient’s resource use and care quality. Although there’ve been abundant empirical studies of the evaluations of DRG-based payment method, China’s health system is different from other countries, which shares various provider incentive mechanisms(13). The payment model in Zhongshan provided us a rather good example to understand how this payment method affects the physicians’ behaviors and health outcomes in China’s health scenario before it is generalized in the whole country.

## Materials and Methods

### Sites and Sampling

A retrospective cohort study among patients with acute myocardial infarction (AMI) from 2008 to 2014 was designed. Two general tertiary hospitals were selected as our study sites. They were the largest local hospitals and treated over 80 percent of all AMI patients in the city. In 2014, 62 percent of all admitted patients and 69 percent of the total inpatient revenues in the two sites were paid through the new DRG-based system.

The disease of AMI has been widely used to evaluate physician’s response to the policy change (14, 15). Hospital treatment of AMI patients includes three approaches: 1) drug therapy; 2) percutaneous coronary intervention (PCI); and 3) coronary artery bypass grafting (CABG). Treatment expenditures vary greatly, with drug therapy being the lowest and CABG the highest. PCI is the primary choice for treating AMI because of its superior clinical effects (16).

In order to study provider’s behavior change before and after the payment reform in 2010, we just focused on patients who received drug therapy or PCI during hospitalization. CABG was excluded in our study because of its too small sample size (15 cases, accounts for 0.44% of the total sample population) and not feasible for statistical analysis. So the inclusion criteria for our study population were:

1) The primary diagnosis was AMI (ICD-10 code: I21.0-I21.4, I21.9);
2) Patients admitted to the study hospitals between Jan. 2008 and Dec. 2014;
3) Patients received drug treatment or PCI procedure during hospitalization.

Prior to the payment reform, hospitals received a flat payment for treating the insured AMI patients, no matter what procedure they received. After the reform, the weights of patients receiving drug therapy rank from 152 to 222, with an average weight of 203; and of patients receiving PCI rank from 502 to 969, with an average weight of 796. The reimbursement price for PCI group is nearly quadrupled the drug group. In this case, physicians would be motivated to provide appropriate treatment for AMI patients. We chose the indicators of the percentage of PCI use and total hospital expenses per admission to measure the impact on patient’s resource use after the reform, and the indicators of in-hospital mortality and re-admission rate within 30 days after discharge to evaluate the impact on quality of care.

### Data Collection and Statistical Analysis

Hospital electronic medical record (EMR) data of AMI patients in the two study hospitals were employed. Information collected included patients’ demographic characteristics, principal diagnosis, admitted and discharged dates and departments, length of stay (LOS), procedure performed, status at discharge, and total hospital expenses. The new DRG based payment system was adopted on July 1, 2010. In our study, patients discharged from Jan. 2008 to Jun. 2010 were considered as the pre-reform period; and from Jul. 2010 to Dec. 2014 the post-reform period. A total of 2895 inpatients were included as our study sample, among which 727 were discharged before the reform and 2168 afterwards.

Fifty one percent of the population in Zhongshan were migrant workers. The social insurance programs in China are city-level managed. The migrants’ medical reimbursement affairs are not charged by the place they work but by where they are from. Given that, patients with insurance scheme in Zhongshan were regarded as the intervention group in our analysis, while patients not covered were as the control group. We assumed the two groups had similar consumer behaviors since their medical costs could be reimbursed by their local social insurance schemes finally.

Difference-in-difference (DID) methodology was used to evaluate the net policy effect (11, 15, 17). Multiple regression analyses were conducted to determine if the implementation of new payment system in Zhongshan was associated with the outcome measures. The model specification was as follows:

$$Y=\alpha +\beta \mathrm{Time}+\gamma \mathrm{Group}+\delta \left(\mathrm{Time}\times \mathrm{Group}\right)+\varphi_{i}X_{i}+ɛ$$

where *Y* represents the outcome variables. In our analysis, the hospital expenditure variable was not normally distributed so it was performed natural log transformation. The amounts discussed herein were estimated based on 1 RMB yuan equals 0.1629 US$. *Time* was a dummy variable that equals 1 if the patient was discharged after the reform and 0 otherwise. *Group* was a dummy variable that equals 1 if the patient was insured and 0 otherwise. *Time* × *Group* was the cross-product of *Time* and *Group* that equals 1 if the patient was insured and hospitalized during the test period. Vector *X_ i_* indicates all other control variables that could potentially affect the outcome variables, including patients’ *age*, *gender* and *LOS*. The variable of *LOS* was abnormally distributed and transformed natural logarithm in regression models.

In the estimation model, (β+δ) indicated changes for the intervention group after the payment reform than before, while β indicated changes for the control group. δ is to measure the changes of the intervention group than the control group after reform, which was interpreted as the net impact of the payment reform in Zhongshan.

All information about hospitals’ and patients’ names or other private information was bleached for analysis. And there were no blood sample or questionnaires used in this study. The Academic Ethics Committee of Public Health and Nursing Research, Shanghai Jiao Tong University considered the research did not have to apply for ethical review.

## Results

### Descriptive statistics

Table 1 shows the baseline characteristics of the sample population. The proportion of the insured patients were similar both before and after reform, which was in line with the population structure in Zhongshan. Two groups before and after the reform also shared the comparable features in age and gender distribution (*P*=0.5894 and 0.9147, respectively). The average length of stay (ALOS) for the post-reform population was significantly decreased by 0.14 days (*P*=0.0010). In terms of the treatment method, drug therapy was the dominant method before reform and PCI only accounted for 10.18 percent. However, after reform the percentage of PCI use sharply increased to 73.80 percent (P<.0001). Meanwhile, the total hospital expenses were significantly increased from $6083.54 to $7032.38 after reform (*P*<.0001). The hospital mortality significantly dropped from 3.03 percent to 1.75% after reform (*P*=0.0438). There was no difference in readmission rates before and after reform in our study (*P*=0.3706).

**Table 1:** Characteristics of patients

***Variable***	***Before reform***	***After reform***	***P value***
No. of patients, n(%)	727 (25.11)	2168 (74.89)	0.9151
Insured	339 (46.63)	1006 (46.40)	
Uninsured	388 (53.37)	1162 (53.60)	
Sex, n(%)			0.9147
Male	566 (77.85)	1692 (78.04)	
Female	161 (22.15)	476 (21.96)	
Age, mean(std)	62.18 (13.27)	61.99 (14.08)	0.5894
ALOS, mean(std)	8.71 (5.38)	8.57 (6.21)	0.0010
Therapy, n(%)			<.0001
Drug	653 (89.82)	568 (26.20)	
PCI	74 (10.18)	1,600 (73.80)	
Total hospital expenses, $, mean (std)	6083.54 (4055.63)	7032.38(6060.44)	<.0001
Died in hospital, n(%) (missing value=343)	22 (3.03)	32 (1.75)	0.0438
Readmission within 30 days, n(%)	15 (2.06)	34 (1.57)	0.3706

ALOS: average length of stay; PCI: percutaneous coronary intervention

Table 2 shows the descriptive analysis and univariate results of the four outcome variables. The results revealed that, after payment reform, PCI use for both two groups were significantly increased by 61.21 and 65.76 percent, respectively (both *P*<.0001). The DID value showed PCI use for the intervention group decreased by 4.55 percent after reform. The total hospital expenses for the intervention group had an insignificant increase of $423.96 after reform (*P*=0.2428), while for the control group had a considerable increase of $1410.06 (*P*<.0001). The DID result suggested a decrease of $986.10 for the intervention group after reform.

**Table 2:** Descriptive and univariate analysis on health usage and quality

***Outcome variables***	***Before reform***	***After reform***	***Diff***	***DID***
**PCI use, %**				
Intervention group	15.93	77.14	61.21 ^***^	−4.55
Control group	5.15	70.91	65.76^***^	
**Total hospital expenses, $, mean(std)**				
Intervention group	6938.05 (3944.06)	7362.01 (4179.60)	423.96	−986.10
Control group	5336.94 (4008.96)	6747.00 (7297.73)	1410.06^***^	
**Hospital mortality, %**				
Intervention group	2.65	1.29	−1.36	−0.33
Control group	3.35	2.32	−1.03	
**Readmission within 30 days, %**				
Intervention group	1.77	1.49	−0.28	0.40
Control group	2.32	1.64	−0.68	

Diff: difference; DID: difference-in-difference; PCI: percutaneous coronary intervention. //

* *P*<0.05;

** *P*<0.01;

*** *P*<0.001

Besides, after reform both two groups of patients had the slight but not significant decreases in hospital mortality (*P*=0.0872 and 0.2979, respectively). The same trend were observed in the re-admission rate variable (*P*=0.7202 and 0.3808, respectively). But the DID results indicated the hospital mortality for the intervention group was decreased by 0.33 percent and the readmission rate was increased by 0.40 percent after the new payment system was implemented. Neither of them had statistical significance in our study.

### Multiple regression results

We further examined the impacts of payment reform on patients’ PCI use and hospital expenses using DID regression models. The results are presented in Table 3 and Table 4. Compared with the control group, there was a significant decrease in PCI use for the intervention group after reform (Odds Ratio [OR]: 0.40; 95% Confidence Interval [CI]: 0.23, 0.72). Male younger patients with longer length of stay were more likely to conduct PCI therapy. Especially, we found the probability of the control group using PCI therapy had increased by 53.91 times after the payment reform. Meanwhile the hospital expenses for the intervention group was significantly decreased after reform (b=−0.15, *P*=0.0037). Male patients with longer length of stay and conducted PCI during hospitalization were associated with higher hospital expenses.

**Table 3:** Multiple regression results of the effects on PCI use

***Variable***	***Estimate***	***P value***	***OR (95% CI)***
Intercept	− 3.97	<.0001	
Time	3.99	<.0001	53.91 (33.50, 86.73)
Group	1.20	<.0001	3.33 (1.94, 5.72)
Time^*^Group	−0.91	0.0020	0.40 (0.23, 0.72)
Age	−0.01	<.0001	0.99 (0.98, 0.99)
Sex (female as reference)	0.61	<.0001	1.84 (1.47, 2.31)
LOS	0.70	<.0001	2.02 (1.73, 2.35)

PCI: percutaneous coronary intervention; LOS: length of stay; OR: odds ratio; CI: confidence interval

**Table 4:** Multiple regression results of the effects on hospital expenses

***Variable***	***Estimate***	***P value***	***95% CI***
Intercept	6.90	<.0001	6.75, 7.05
Time	−0.32	<.0001	−0.40, −0.24
Group	0.24	<.0001	0.15, 0.33
Time^*^Group	−0.15	0.0037	−0.25, −0.05
Age	0.002	0.4889	−0.004, 0.000
Sex (female as reference)	0.06	0.0377	0.00, 0.12
LOS	0.60	<.0001	0.56, 0.63
Therapy (drug as reference)	0.85	<.0001	0.81, 0.89

PCI: percutaneous coronary intervention; LOS: length of stay; OR: odds ratio; CI: confidence interval

* *P*<0.05;

** *P*<0.01;

*** *P*<0.001

## Discussion

Abundant evidence shows physicians respond sensitively to hospital payment policies and adjust their clinical behaviors actively to hospital payment changes (13, 18). Our study investigated the impacts of the innovative DRG-based payment with global budget in Zhongshan. The results suggested it had a positive effect on AMI patient’s cost containment but negative effect on encouraging resource use. The new payment method had no significant impacts on patients’ care quality.

As mentioned before, PCI was a major procedure for treating the disease of AMI. Our results showed an incredible increase of over 60 percent in PCI use for both two groups after reform. This huge increase is mainly attributable to the technology progress of cardiac interventional therapy in China. The past decade had witnessed a fast increase of PCI use in this country, with an annual growth rate of 16 percent. It is reported that the number of PCI cases in China in 2017 was 1.1 million, which was nearly 5 times than that in 2009 (19). Nevertheless, our DID results revealed a significant decrease in PCI use for the intervention group. A potential explanation is that the physicians’ intention to gain profit through providing PCI service to the insured had diminished after reform.

Coincidently, we found a considerable decrease of total hospital expenses for the insured patients in our study. This result was consistent with our study in Shanghai (20) and studies in other countries (21, 22). But some studies found the opposite results of increasing costs after DRG was implemented (23, 24). The effect of DRG payment system on containing costs per discharge is mixed. More evidence is needed for China in the further to monitor its effects.

Additionally, we observed a remarkable increase in hospital costs for the uninsured patients. The similar result was observed in another study in Shanghai DRG payment reform (25) but not observed in Beijing reform (11). The cost shifting consequence from the insured to the uninsured under the DRG-based payment system should be noted in future payment mechanism design.

Our study suggested no significant changes in AMI patients’ hospital mortality or readmission rate within 30 days for both two groups after reform. Literatures about quality evaluations of DRG-based hospital payment reform found confounded results in quality evaluation (4). Besides, how to explain the mechanism of hospital payment reform on patient’s quality outcomes remains unclear. More studies in this area are of vital importance for both researchers and policy makers in China.

After years of pilots, the State Council announced to adopt the DRG-based prospective system to replace the current retrospective payment system in all public health facilities in 2020 (26). This paper was a case study of comparing the DRG-based budget payment with a unique capitation-like payment in China. As we all know, fee-for-service is the prevailing method to pay for hospitals in this country and has been discussed a lot (27, 28). However, there also exist some other payment methods in local practices. For example, among 18 cities in Guangdong province where we studied, 13 of them adopted the capitation-like payment. So case studies of comparing the DRG payment with various kinds of existing payment methods are necessary to provide evidence for the governments’ policy making. Also, China will provide rich experiences to share with other countries.

### Limitations of the study and directions for future study

There were several limitations in this paper. First, only AMI patients were included in our study. Physicians’ responses to hospital payment change may vary among different DRGs. If possible, samples of other DRGs, especially the surgical DRGs, should be used for further investigation. Second, some of the patient’s social demographic factors were missed, which might cause bias in the regression analysis. Lastly, if possible, the analysis from the provider’s perspective could be conducted to provide more evidence of how the payment policy change affect physician’s behaviors.

## Conclusion

The DRG-based payment with global budget in Zhongshan demonstrated a positive effect on AMI patient’s cost containment but negative effect on promoting resource use. No evidence was observed of the payment reform with patient’s quality of care. However, cost-shifting to the un-insured should be paid attention in payment reform implementation in China. More evidence of the impacts of the DRG-based payment in different regions is needed before it is generalized.

## Ethical considerations

Ethical issues (Including plagiarism, informed consent, misconduct, data fabrication and/or falsification, double publication and/or submission, redundancy, etc.) have been completely observed by the authors.
